# Seven unconfirmed ideas to improve future ICU practice

**DOI:** 10.1186/s13054-017-1904-x

**Published:** 2017-12-28

**Authors:** John J. Marini, Daniel De Backer, Can Ince, Mervyn Singer, Frank Van Haren, Martin Westphal, Paul Wischmeyer

**Affiliations:** 1 University of Minnesota, Minneapolis, MN USA; 2 Department of Intensive Care, CHIREC Hospitals, Université Libre de Bruxelles, Brussels, Belgium; 3 Department of Intensive Care, Erasmus MC, University Medical Center Rotterdam, ‘s-Gravendijkwal 230, 3015 CE Rotterdam, The Netherlands; 4 Department of Translational Physiology, Academic Medical Center, University of Amsterdam, Meibergdreef 9, 1105 AZ Amsterdam, The Netherlands; 5 Bloomsbury Institute of Intensive Care Medicine, University College London, London, UK; 6 University of Canberra, Canberra, Australia; 7 Australian National University, Canberra, Australia; 8 Intensive Care Unit, Canberra Hospital, Canberra, Australia; 9 Department of Anesthesiology, Intensive Care and Pain Medicine, University of Muenster, Muenster, Germany; 10 Department of Anesthesiology and Surgery, Duke Clinical Research Institute, Duke University School of Medicine, Durham, NC USA

**Keywords:** Microcirculation, Resuscitation, Shock, Sepsis, Ventilator-induced lung injury, Personalized medicine, Melatonin, Adaptive clinical trials, Metabolic monitoring

## Abstract

With imprecise definitions, inexact measurement tools, and flawed study execution, our clinical science often lags behind bedside experience and simply documents what appear to be the apparent faults or validity of ongoing practices. These impressions are later confirmed, modified, or overturned by the results of the next trial. On the other hand, insights that stem from the intuitions of experienced clinicians, scientists and educators—while often neglected—help place current thinking into proper perspective and occasionally point the way toward formulating novel hypotheses that direct future research. Both streams of information and opinion contribute to progress. In this paper we present a wide-ranging set of unproven ‘out of the mainstream’ ideas of our FCCM faculty, each with a defensible rationale and holding clear implications for altering bedside management. Each proposition was designed deliberately to be provocative so as to raise awareness, stimulate new thinking and initiate lively dialog.

## Background

In our current era of evidence based medicine, empirical results of clinical trials are most highly valued as the foundation of our knowledge, while experience-based and eminence-based opinions, often published as commentaries, editorials, essays and letters, take a ‘back seat’. Yet, some insights that stem from the intuitions of experienced clinicians, scientists and educators help place current thinking into proper perspective and point the way toward formulating the novel hypotheses that direct future investigative scientific efforts. In fact, while technical innovations, experimental observations and statistical meta-analyses often lead clinical practice, at other times the opposite is true; conflicting data from imperfect studies generate lingering confusion and doubt. With imprecise definitions, study executions and measurements, our clinical science often lags behind practical experience and simply documents what appear to be the faults or validity of ongoing practice. As we have experienced in recent decades, progress made with these blunt tools for advancing the care of the individual can be vexingly slow and inexact. Better study designs, targeted biomarkers, integrative as opposed to reductionist thinking and ‘big data’ capabilities hold genuine promise to personalize critical care—but clearly, we are not there yet.

In most meeting formats, faculty presenters rely on established principles and an examination of what has recently been published or confirmed to present their ideas. Debates and panel discussions either help bring about consensus (always comforting and sometimes dangerous) and/or highlight genuine differences of opinion. As in the prior two Future of Critical Care Medicine (FCCM) meetings, one of our sessions took a radical departure from those traditional approaches. What follows is a wide-ranging set of unproven ‘out of the mainstream’ ideas of our faculty, each with a defensible rationale and holding clear implications for altering bedside management. The intent of this provocative format was to stimulate thinking and interchange, and perhaps to point toward new directions for productive research, concept development, and eventual application to improved care for the critically ill.

## Daniel De Backer: Release tissue nitric oxide for improving microvascular perfusion

### Background

Tissue perfusion can be altered even when cardiac output and arterial pressure remain within reasonable goals. Alterations in microvascular perfusion have been shown to occur in sepsis and septic shock [1], as well as in a variety of other conditions (cardiogenic shock, trauma, ischemia reperfusion injury, etc.). These are characterized by the close proximity of non-perfused vessels to perfused vessels, leading to microvascular shunting and increased oxygen diffusion distances. The severity and the duration of such microcirculatory disturbances relate to mortality and development of organ dysfunction [2]. Several mechanisms have been implicated in the development of these alterations, including loss of communication between vascular segments, impaired endothelial reactivity, alterations in red and white blood cell rheologies, alteration in endothelial glycocalyx, platelet aggregation and microthrombosis.

Given the characteristics of microvascular alterations and the mechanisms potentially implicated, it seems logical to try to recruit the microcirculation more than to increase the flow within the already perfused vessels. In addition, an ideal intervention should help reverse the implicated pathological mechanisms, not only improve microvascular perfusion.

Among the suggested interventions, certain vasodilatory agents have been proposed. Nitroglycerin was the first to be introduced. After initial promising results of a pilot trial including eight patients [3], a confirmatory randomized trial found no difference in the changes in microvascular perfusion between patients receiving nitroglycerin and placebo. Several factors may explain this negative result, including a potential decrease in nitric oxide (NO) generation from nitroglycerin, as it requires efficient aldehyde dehydrogenase type 2 that may be inhibited in sepsis [4].

Another point of attack is to exploit local release of NO at the microcirculatory level to promote targeted vasodilation. There are two known ways to boost NO at the endothelial level, one through endothelial NO synthase and the other through nitrite reduction.

For the first reaction (Arginine + O_2_ = > Citrullin + NO], oxygen and an effective endothelial NO synthase are required. Interestingly, endothelial NO synthase may be dysfunctional in sepsis or in ischemia/reperfusion injury, due to a decrease in one of its mandatory cofactors, tetrahydrobiopterin. In an ovine model of septic shock, administration of tetrahydrobiopterin improved microvascular perfusion, vascular permeability, organ function and survival time [5]. Similarly, administration of vitamin C, which increases tetrahydrobiopterin availability, also improves microvascular perfusion in sepsis, in a pathway that depends on endothelial NO synthase [6].

The second reaction converts nitrite into NO (Hb[Fe^2+^] + NO_2_^−^ + H^+^ => NO + Hb[Fe^3+^] + OH^−^). This reaction is accelerated in the presence of deoxyhemoglobin, as opposed to oxyhemoglobin [7]. As a result, it mostly occurs in the microcirculation in the hypoperfused capillaries of metabolically active areas where oxygen saturation is low.

### Idea

Administer nitrites with the hope that they could be converted into NO in the most vulnerable parts of the microcirculation.

## Can Ince: Better resuscitation fluids for shock can be devised that improve perfusion, boost oxygen delivery and reduce inflammation

### Background

Resuscitation from cardiocirculatory compromise is aimed at correcting decreased microcirculatory perfusion by improving blood flow and consequently sustaining tissue oxygenation. Shock is associated with a compromise in oxygen transport to the tissues, resulting in organ dysfunction. If left uncorrected this condition results in organ injury and, ultimately, in organ failure. The current approach to resuscitation is to target systemic perfusion by administration of vasoactive compounds and non-oxygen carrying salty solutions with or without larger colloid molecules to ensure a longer presence in the circulation.

The primary challenge in fluid therapy is to ensure that sufficient oxygen gets transported to the microcirculation and ultimately to the tissue cells. Here conventional fluids fail on two counts: The first shortcoming of conventional fluids is their hemorheological effect. Lower viscosity reduces the ability of the diluted blood to recruit unfilled capillaries (for which the viscosity of hematocrit is needed). This reduces the functional capillary density, creating larger diffusion distances between oxygen-carrying red blood cell-filled capillaries and the respiring cells of organ tissue. The second potential shortcoming of resuscitation fluids is their poor oxygen solubility (less than 3% compared to hemoglobin (Hb)), which decreases the oxygen carrying capacity of blood. Indeed, several experimental studies directly measuring microcirculatory oxygen availability following fluid resuscitation in models of shock have shown repeatedly that although fluids are capable of correcting systemic hemodynamic variables such as blood pressure and cardiac output, they can be ineffective in improving microcirculatory perfusion and tissue oxygenation in vulnerable organs such as the kidney. The only therapeutic modality to improve oxygen levels in the microcirculation is provided by blood transfusions [8]. However, there is much clinical reluctance to administer blood due its potential harmful side effects, such as the rise in free Hb and the potential immunological response of the host to homologous blood transfusions.

An alternative to homologous blood transfusion is offered by Hb-based oxygen carriers (HBOCs), and many such compounds have been developed over the past decades. Experimentally these compounds have been shown to improve microcirculatory oxygen availability in models of shock [9]. Although conceptually appealing, their clinical introduction has been mired by problems. These include the vasopressor effect caused by their high affinity for NO, causing vasoconstriction. Different types of HBOCs have been developed with the aim of reducing this vasopressor effect, but these compounds have not shown clinical efficacy. The reason for this lack of success are several; a) at the bedside, there is an inadequate monitoring methodology to assess the need for tissue oxygenation to indicate the need for HBOCs over simply giving fluids; b) the p50s of HBOCs ranges from 5 mmHg (where oxygen will stay stuck to the Hb) to p50s in the range of that of natural Hb where oxygen can be easily lost and not reach the parenchymal cells in need of oxygen; c) inadequately designed clinical trials have generated little  knowledge about when, how much and to what target.

From a different perspective, HBOCs conventionally have been administered as stand-alone drugs, whereas they could potentially be used as an adjunct to conventional fluid therapy to increase the oxygen-carrying capacity of volume therapy. Besides improving perfusion and oxygenation, resuscitation fluids should also be effective in reducing and controlling inflammation. In pursuit of this idea, investigations have been conducted to examine the potential anti-inflammatory and anti-oxidant effects of infused fluids (e.g., starches) as well as attaching molecules such as CO and glutathione to HBOC to control inflammation and oxidative stress [10].

From a therapeutic hemodynamic point of view, therefore, resuscitation requires reversal of shock, sepsis and hypovolemia. The first objective is to ensure microcirculatory and tissue perfusion; second, to restore perfusion is accompanied by a restoration of adequate tissue oxygen availability; and finally, to ensure that the cellular constituents of the microcirculation and parenchymal cells are protected from injury associated with the inflammatory molecules and oxidative and nitrosative stress associated with shock and reperfusion.

The two categories of resuscitation fluid in current use are blood and non-oxygen carrying crystalloid or colloid solutions. Each of these has been shown to have adverse effects due to composition and uncertainty about how to titrate these solutions under different conditions of hypovolemia and shock, where too much and too little are both considered harmful. As already mentioned, the ultimate target for administering the optimal volume resides in the microcirculation for which handheld microscopy could be a potential tool to optimally administer resuscitation [11]. However, fluid content needs also to be more personalized, matching the specific needs of individual patients.

In doing so, a broth of molecules must be configured that addresses the specific type of fluid needed to target the specific physiological compartment of the individual patient in need of resuscitation. The compartments which can be identified include intracellular, interstitial and intravascular. Intracellular hydration requires a crystalloid solution with glucose, whereas interstitial hydration needs any physiologic crystalloid solution. Intravascular hypovolemia is best addressed by a colloid solution to ensure sustained filling of the vasculature. Resuscitation fluid should ideally also carry anti-inflammatory and oxygen carrying agents to completely meet the goals of resuscitation.

When considering the ultimate composition of such an ideal resuscitation fluid, one is reminded of the beneficial effects of the chicken soup cure for common inflammatory conditions [12]. It carries nutrients, provides hydration, is isotonic (if you don’t add salt of course) and is anti-inflammatory. Looking at chicken soup as a pharmacological agent for resuscitation, therefore, it seems like an ideal candidate. What is missing in chicken soup, however, is an oxygen-carrying agent such as a HBOC. Such a ‘pink chicken soup’ would hydrate, fill the vasculature (colloid effect), offer anti-inflammatory properties and promote the transport of oxygen to the tissues (the pink stuff).

### Idea

A ‘pink chicken soup’ that addresses the deficiencies of our current options may indeed be a candidate for the optimal shock resuscitation fluid of the future.

## John J. Marini: We should address the forgotten but crucial vascular side of ventilator-induced lung injury

### Background

Although ventilator-induced lung injury (VILI) is undoubtedly a complex process that is influenced by many factors, the great majority of investigative attention has been directed to airspace mechanics, as exemplified by tidal volume, plateau pressure, PEEP and driving pressure. Yet, because the fragile alveolus serves as the interface between gas and blood, and because the stresses applied to the airway epithelium also impact vascular endothelium, the potential for vascular pressures and flows to impact the development and/or evolution of VILI also deserves close consideration.

Mechanical forces that tear the delicate alveolar-capillary membrane can originate on either side of the boundary. Adverse ventilatory patterns applied to previously healthy lungs not only cause proteinaceous edema, but also neutrophil aggregation and hemorrhage [13]. In the supine position, hemorrhagic edema forms preferentially in dependent areas [14, 15]. This proclivity is not subtle, and has been corroborated by the work of investigators using diverse injury models [16]. The tendency for hemorrhage to occur preferentially in the most dependent regions of the lung may have several explanations. One compelling reason to expect microvascular disruption to occur there is that the mechanical stresses applied by the tidal inflation cycle are greatly amplified at the interface of opened and closed lung tissues. An admittedly oversimplified geometrical argument indicates that strains at high airway pressure are several times as great as that experienced in the free walls of the open alveolus.

It is somewhat counter-intuitive that tissue disruption should occur in areas in which transmural stretching forces (as defined by plateau pressure minus pleural pressure) are weakest. That is to say, “alveolar stretch” is greatest in the non-dependent regions, which are relatively spared both the hemorrhagic infiltrate and most signs of inflammation. The tendency for hemorrhage to occur preferentially in the most dependent regions of the lung may have several explanations. The local mechanical driving power [17] may far exceed that experienced in non-dependent zones. Although understudied, surfactant depletion and inflammatory weakening of the interstitial structure could amplify the impact of such forces, whereas other changes of the microenvironment (e.g., flooding by edema) could abrogate the mechanical stresses experienced in distal lung units.

Interactions between vascular pressure and ventilation suggest strongly that closer attention should be paid to interventions that impact vascular pressures, flows, and resistances when high inflation pressures are in use. Because microvascular stresses appear to be a potent cofactor in the development of pulmonary edema as well as lung damage resulting from an injurious pattern of ventilation, the clinician managing acute lung injury must reconcile the competing objectives of ensuring adequate oxygen delivery and minimizing adverse effects. If increased pre-alveolar microvascular pressure accentuates a tendency for VILI, attempts to raise cardiac output may have unintended consequences. On the other hand, taking steps to reduce oxygen consumption demands could benefit the lung by reducing the pressure gradient developed across the microvasculature. Reduction in the demands for cardiac output and ventilation could dramatically reduce the tendency for VILI even when using patterns that generate similar values for peak, end-expiratory, and driving alveolar pressures.

### Idea

Restrain pulmonary vascular flows and pressures by lowering oxygen demand to further reduce the incidence and severity of VILI.

## Mervyn Singer: Outcomes and therapeutic responses to ICU care can be predicted for septic patients

### Background

Septic critically ill patients may be predestined to survive or die, perhaps explaining the failure of the many trials testing interventions to interrupt the natural course of sepsis. Indeed, we have not shown yet that we can “beat nature”; most progress over the past 20 years relates to inflicting less iatrogenic harm to the patient, not improving response and accelerating natural healing. If this analysis is correct, we inadvertently may simply prolong the course to death among those destined not to survive, at high personal and economic costs.

Using modern bioanalytical techniques, numerous studies have demonstrated the predictive potential of biomarkers, metabolomes, and proteomes to differentiate among eventual survivors and non-survivors of sepsis, even at a very early stage. Three interesting studies conducted from blood samples taken upon presentation to the emergency room have demonstrated that inflammatory cytokines (IL-6, IL-10) [18], cardiac troponin-T [19], and metabolomes characterizing fatty acid transport, gluconeoenesis, and the citric acid cycle [20] can point the way to the outcome that time would eventually reveal.

Similar prognostication regarding the likely response to treatment is also possible using both inflammatory biomarkers or physiological indicators. For example, stratification on the basis of inflammatory biomarkers may help direct immune modulatory therapy. Steroids given to septic animals predicted to die proved beneficial; steroids given to those projected to survive did not [21]. Reflections of these experimental observations were observed retrospectively using physiological parameters in the CORTICUS hydrocortisone trial [22]. Despite the increased risks of superinfection that the corticosteroids imposed, those septic patients with systolic blood pressures persisting below 90 mmHg after one day of appropriate fluids and vasopressors experienced a significant reduction of mortality risk if given hydrocortisone.

Another important controversy regarding pharmacotherapy of sepsis concerns the wisdom of beta blockade. In a randomized clinical trial, esmolol showed the distinct potential to reduce mortality risk, but only for patients who were both tachycardic and receiving high-dose norepinephrine therapy at 24 hours, reflecting a similar result as obtained with experimental sepsis in rats [23]. In rats with septic physiology, esmolol treatment appeared to benefit animals predicted not to survive and to harm the predicted survivors.

Such examples show that using both physiological and molecular indicators may allow us to predict the eventual outcome of life-threatening sepsis at an early time point and thereby select our treatments more effectively. Such predictive ability would not only improve the design of our clinical trials, but also help to select therapies for the individual that hold the most potential for effective and humane care.

### Idea

Predict potential survivors and non-survivors of sepsis causing critical illness at an early stage and treat these patient categories differently.

## Frank Van Heren: Novel adaptive designs of clinical trials improve their efficiency and value

### Background

In clinical research, randomization among alternatives is central to progress because associations and inferences from observational studies may not prove causative. Unfortunately, as currently conducted, our large randomized trials often conflict and generally have proven disappointing in the critical care setting [24]. The most likely explanations include imprecise definitions, inexact or inappropriate controls, and an inability to control or account for all influential variables, as important synergistic interactions produce emergent phenomena that are not accounted for in the trial design. Inability to recruit sufficient numbers of appropriate candidate patients over a reasonable time drags out the data collection process (often attenuating relevance to current practices) or terminates many such investigative efforts.

One innovative approach to randomized trial design is to depart from rigid one-to-one randomization and into adaptive allocation to the study limbs in accordance with relative response as the study progresses. Under this paradigm, if a subgroup starts to do better with one treatment, more future patients are allocated to that limb to confirm or refute that trend and accelerate the pace of the investigation. Frequent looks at the developing data are implicit when taking this approach.

The platform trial, an efficient strategy for simultaneously and sequentially evaluating numerous treatments within the framework of a single study, has been proposed by Berry and colleagues [25] as a tool with which to determine their relative worth among a heterogeneous population. This approach recognizes the imprecision of our current definitions and classifications, as it explicitly recognizes that targeted populations and treatment responses may be heterogeneous, even when careful measures are taken to be appropriately selective. Such a strategy departs from that of the traditional trial, which assumes itself to be testing the efficacy of a single intervention in a generally homogeneous population. A unique aspect of this particular “adaptive” approach is that the platform trial can be carried out over the long-term—even perpetually, so long as there are suitable treatments requiring evaluation. The number of treated groups or specific treatments may change over time, with specific individual treatment groups removed for demonstrated efficacy or harm. Such capability departs from our current “fixed randomization” approach in which the entire trial is stopped for success, futility, or harm based on the effects of a single experimental treatment. We must change our clinical trial paradigm so that we recognize current limitations. Should we embrace the principle that most major public health problems should be the subject of perpetual global adaptive trials?

### Idea

Continuous and adaptive clinical trials with interchangeable parts should be the research standard.

## Martin Westphal: Disruption of sleep patterns and circadian rhythms is an important and addressable cause of varied ICU morbidities

### Background

Melatonin, a hormone produced in the pineal gland, is well-known to influence the state of wakefulness. It is synthesized and released under the regulation of the clock genes concentrated and expressed in the suprachiasmatic nucleus of the brain. Promoted by darkness, release of melatonin peaks during health in the early morning hours and plummets just before awakening. In the ICU, that natural diurnal rhythm is seriously disrupted by internal factors related to the critical illness as well as by external factors such as light, noise, continuously infused sedatives, stress, and varied medical treatments [26]. Disrupted sleep architecture and cumulative sleep deficits are contributors to lingering delirium [27], now thought to be a major contributor to delays in extubation and rehabilitation. Selected clinical trials have shown the ability of exogenously administered melatonin to help address these issues [28]. In concept, better sleep should speed recovery and help prevent the post-ICU syndrome.

Less well appreciated by intensive care unit practitioners is the intriguing body of research evidence indicating that melatonin may have other important roles to play during serious illness and recovery. The spectrum of melatonin’s effects unrelated to sleep ranges from antioxidant properties to antimicrobial activity and immunomodulation [29]. Neuroprotective [30], antioxidant, infection inhibiting, and anti-neoplastic actions have been reported [29]. Given melatonin’s central importance as a regulatory hormone, restoring its normal daily influence and timing of its concentration could make a major contribution to ICU care. Melatonin plays a pivotal role in the regulation of circadian rhythm, not only for the brain, but for the activities of other vital organs as well. Virtually free of deleterious side effects, melatonin is inexpensive to administer and offers a variety of potentially beneficial actions. It should therefore be routinely used in both the acute and chronic phases of serious illness.

### Idea

Melatonin should be administered to all critically ill patients able to receive it.

## Paul Wischmeyer: Non-invasive metabolism monitoring aids in personalizing critical illness interventions

### Background

Prediction, early intervention, and monitoring of progress are essential to personalize critical care management. Treatments and doses applicable to one stage or severity of critical illness may be inappropriate to apply at a later one. An important challenge is to monitor the patient’s pre-illness, acute, chronic, and recovery stage and status by noninvasive means. Metabolic changes reflect breakdown, rebuilding, and innate cellular functioning during the illness course. One currently available methodology determines the relationship of two carbon isotopes in exhaled gas to determine stage and status: the ^13^CO_2_/^12^CO_2_ ratio [31]. C^12^, the far more common form in the healthy individual, is easier to break down than C^13^ for energy production, and C^12^ is freely exhaled as ^12^CO_2_. In contrast, C^13^ is incorporated into acute phase proteins and is not exhaled at usual concentrations during the inflammatory state. Consequently, at the very onset of an acute inflammatory illness, the ratio of ^13^CO_2_/^12^CO_2_ decreases in the exhaled gas [32]. In fact, detectable changes in the ratio *precede* the clinically detectable signs of infection. While individual point determinations of breath analyses do not correlate well with the inflammatory status, *trends* in the ^13^CO_2_/^12^CO_2_ ratio clearly do.

In clinical studies, down-trending of the ^13^CO_2_/^12^CO_2_ ratio in exhaled gas significantly precedes alterations in leukocytes, body temperature, and clinical suspicion of such developing infections, such as pneumonia. Conversely, reversal of the trend in the ratio indicates that inflammation is receding and that a stage transition is underway, perhaps indicating the emergence of different nutritional requirements. Such trend analyses appear to be both sensitive and specific. Other potential applications of the ^13^CO_2_/^12^CO_2_ ratio monitoring include determination of underfeeding or overfeeding status.

Metabolism monitoring may hold therapeutic potential not only for the acute state but also for the rehabilitation phase [33]. It is known that healthy mitochondria primarily utilize fatty acids to produce energy. In conjunction with the other standard physiological and biochemical indicators, monitoring of fat oxidation rate versus carbohydrate oxidation rate may help characterize tissue vitality and rehabilitation progress, and thereby help shape targeted exercise/nutritional/hormonal programs to aid faster recovery from critical illness.

### Idea

Metabolism monitoring should be used to diagnose infection, determine the phase of critical illness, and guide post-ICU rehabilitation.

## Conclusion

The seven ideas presented here are intended to be conceptually provocative and are not yet grounded in definitive scientific evidence. Nonetheless, the authors hope that they provide points of departure for thought, discussion and future investigation.
